# Predicting Failure of Conventional Imaging in Primary Hyperparathyroidism: The Impact of Parathyroid Hormone Levels and Thyroid Nodules

**DOI:** 10.5152/eurasianjmed.2026.261492

**Published:** 2026-08-12

**Authors:** Ceren Erdoğan Eroğlu, Şeyma Açık, Işılay Kalan Sarı

**Affiliations:** Department of Endocrinology and Metabolism, Antalya Training and Research Hospital, University of Health Sciences, Antalya, Türkiye

**Keywords:** Localization failure, parathyroid scintigraphy, primary hyperparathyroidism, ultrasonography

## Abstract

**Background:**

Primary hyperparathyroidism (PHPT) is an endocrine disorder caused by excessive parathyroid hormone (PTH) secretion. Accurate localization remains essential for the clinical evaluation of patients; however, successful localization cannot be achieved in all patients. This study aimed to evaluate clinical and biochemical factors associated with unsuccessful localization using conventional imaging in patients with PHPT.

**Methods:**

This retrospective cross-sectional study analyzed data from 174 patients diagnosed with PHPT between January 1, 2015, and October 1, 2024. Localization was performed using technetium-99m MIBI scintigraphy and ultrasonography; fine-needle aspiration with PTH washout was used selectively. Patients were grouped as localized (n = 127) or non-localized (n = 47) according to conventional imaging findings. Demographic characteristics, biochemical parameters, imaging results, and clinical findings were compared.

**Results:**

The localized group had significantly higher intact PTH (iPTH) levels (143.0 vs 115.0 pg/mL, *P* < .001) and serum calcium levels (11.6 vs 11.2 mg/dL, *P* = .004). The non-localized group had higher age (*P* = .012), higher 25-hydroxyvitamin D levels (21.8 vs 17.0 ng/mL, *P* = .024), and more frequent thyroid nodules (74.5% vs 54.3%, *P* = .016). In multivariable analysis, iPTH level (OR: 1.011, 95% CI:1.002-1.020, *P* = .021) and thyroid nodules (OR:0.391, 95% CI:0.174-0.879, *P* = .023) were identified as independently associated with localization success. Receiver operating characteristic (ROC) analysis showed moderate discriminative ability of iPTH cut-off: 146.5 pg/mL (area under the curve = 0.693, sensitivity = 45.7%, specificity = 87.2%).

**Conclusion:**

Higher serum iPTH levels were associated with successful localization, whereas the presence of thyroid nodules was associated with unsuccessful localization using conventional imaging. These findings may help identify patients who require more cautious interpretation of conventional imaging results and individualized consideration of additional imaging modalities.

**Main Points**

- Lower intact parathyroid hormone (PTH) levels and the presence of thyroid nodules are associated with unsuccessful localization using conventional imaging in primary hyperparathyroidism.
- Intact PTH level was independently associated with localization success, although its discriminative performance was moderate.
- Conventional imaging findings should be interpreted cautiously in patients with concomitant thyroid nodules, and additional imaging modalities may be considered on an individualized basis.

## Introduction

Primary hyperparathyroidism (PHPT) is an endocrine disorder that develops as a result of autonomous, excessive parathyroid hormone (PTH) secretion from 1 or more parathyroid glands. Its incidence increases with age and is 3-4 times more common in women than in men.^1^ In a population-based study, the annual incidence of PHPT was reported to be 4 to 6 per 10 000 person-years, with an overall prevalence of 0.84%.^2^^,^^3^

In 80-85% of cases, PHPT is caused by a single parathyroid adenoma, whereas multiglandular disease and parathyroid carcinoma are less common etiologies.^4^ The disease can present with classic or non-classic symptoms. Classic symptoms manifest in bones (bone pain, brown tumors, cysts), kidneys (nephrolithiasis, nephrocalcinosis), and muscles (proximal myopathy). Non-classic symptoms related to cardiovascular, neuropsychiatric, and gastrointestinal systems may also be observed.^5^

The diagnosis of PHPT is established by the presence of elevated or high-normal serum calcium levels along with elevated serum PTH levels or normal PTH levels that are inappropriate for the hypercalcemia.^1^ The definitive treatment for PHPT is parathyroidectomy, and surgical treatment is recommended according to the indications specified in international guidelines.^6^ Accurate localization of pathological parathyroid tissue is essential for optimal clinical management and treatment planning. Ultrasonography (USG) and technetium-99m-methoxyisobutylisonitrile (Tc99m-MIBI) parathyroid scintigraphy performed with single-photon emission computed tomography/computed tomography (SPECT/CT) are the two most commonly used modalities for parathyroid localization in PHPT and provide high sensitivity and specificity.^7^ Another method used for localization is ultrasound-guided fine-needle aspiration (FNA) biopsy of suspicious lesions with PTH washout.

However, successful localization cannot be achieved in all patients with these imaging methods. Negative or discordant imaging findings complicate clinical decision-making and patient management and may require additional imaging studies. Although several studies have evaluated biochemical factors associated with successful localization, the combined influence of biochemical disease severity and concomitant thyroid pathology on localization outcomes has not been fully clarified. Identifying factors associated with successful parathyroid localization and recognizing patients at risk for failed localization may help improve patient stratification and optimize imaging strategies in PHPT. Therefore, this study aimed to evaluate clinical and biochemical factors associated with unsuccessful localization using conventional imaging in patients with PHPT.

## Material and Methods

This retrospective cross-sectional study analyzed data from patients diagnosed with PHPT at the Department of Endocrinology and Metabolism between January 1, 2015, and October 1, 2024. A total of 174 patients aged 18 years and older with a diagnosis of PHPT were included in the study.

Inclusion criteria comprised the presence of PHPT diagnosis, complete availability of parathyroid scintigraphy and neck USG results, data related to variables planned for evaluation, and presence of parathyroid adenoma detected in postoperative pathology results in the localized group. Exclusion criteria included advanced chronic kidney disease (GFR <30 mL/min/1.73m²), secondary/tertiary hyperparathyroidism, normocalcemic hyperparathyroidism, familial hypocalciuric hypercalcemia, multiple endocrine neoplasias, pregnancy/lactation, previous neck surgery/irradiation, medications affecting calcium metabolism (lithium, thiazide diuretics), and incomplete data.

Demographic data, biochemical parameters (serum PTH, albumin-corrected calcium, phosphate, 25-hydroxyvitamin D (25OHD), 24-hour urine calcium, alkaline phosphatase, glomerular filtration rate), imaging results (neck USG, parathyroid scintigraphy, dual-energy X-ray absorptiometry (DEXA), and urinary USG), and PTH washout levels obtained from suspicious lesions when performed were recorded from the hospital information management system. Accompanying thyroid diseases (thyroid nodule, hypothyroidism, hyperthyroidism) were also documented.

Biochemical tests were performed using standard automated analyzers (Beckman Coulter AU5800 and DxI800, Beckman Coulter Inc., CA, USA). If the patient’s albumin was low, corrected calcium was calculated as follows: [Corrected Ca(mg/dL) = measured Ca(mg/dL) + 0.8 × (4 − patient albumin)]. GFR was calculated using the Chronic Kidney Disease Epidemiology Collaboration (CKD-EPI) equation method. Bone mineral densities were measured using DEXA at the lumbar spine (L1-L4) and femoral neck.

Elevated serum calcium levels and serum iPTH levels above the laboratory normal range, in the high-normal range, or inappropriate for hypercalcemia were considered as criteria for the diagnosis of PHPT.^1^ Patients with a history of urinary stones and/or stones detected on USG were also considered as positive for stones.

Neck ultrasonography was performed using high-resolution ultrasound equipment by experienced radiologists. Parathyroid scintigraphy was performed using dual-phase Tc99m-MIBI imaging combined with SPECT/CT. Localization was considered successful if a suspicious parathyroid lesion was identified on Tc99m-MIBI scintigraphy and/or confirmed by positive FNA-PTH washout in cases with suspicious ultrasonographic findings. Fine-needle aspiration–parathyroid hormone washout was performed in selected patients with suspicious lesions detected on ultrasonography, particularly when scintigraphy was negative or when additional confirmation was considered necessary due to discordant imaging findings or the presence of concomitant thyroid nodules. Cases with positive PTH washout results were also considered localized. Patients with PTH washout levels higher than the concurrent serum iPTH level obtained on the day of the procedure were evaluated as positive.^8^ Patients were divided into two groups based on localization status. For this study, localization status was defined according to conventional imaging findings (neck USG, Tc99m-MIBI scintigraphy, and, when necessary, FNA-PTH washout). Biochemical parameters, including serum PTH levels, were not used in determining localization status.

The localized group (n = 127) included patients for whom localization was achieved by parathyroid scintigraphy and/or PTH washout and whose parathyroid adenoma was confirmed by postoperative pathological diagnosis. The non-localized group (n = 47) comprised patients in whom both parathyroid scintigraphy and neck USG were negative, or patients in whom suspicious lesions were detected on neck USG but PTH washout results were negative. Because surgery was not performed in the ­non-localized group, histopathological confirmation was unavailable in these patients.

This study was approved by the Clinical Research Ethics Committee of University of Health Sciences Antalya Training and Research Hospital on October 10, 2024 (reference number: 2024-322). Informed consent requirement was waived due to the retrospective nature of the study. Patient personal data were protected.

This study used ChatGPT to improve language and grammar during the preparation of the manuscript. However, all content of the study has been reviewed and edited by the authors following the use of AI/LLM tools.

### Statistical Analysis

The normality of data distribution was assessed using the Kolmogorov–Smirnov test. For normally distributed variables (phosphate, GFR, and age), independent-samples *t*-test was used; for non-normally distributed variables (calcium, 25OHD, PTH, urine calcium, and ALP), Mann–Whitney *U-*test was applied. Categorical variables were compared between groups using the chi-square test or Fisher’s exact test, as appropriate. All normally distributed variables are expressed as mean ± SD, while non-normally distributed variables are expressed as median (minimum-maximum).

Multivariable logistic regression analysis was performed to determine the factors affecting localization success. Variables with *P *< .05 in univariate analyses were included in the model. Multicollinearity among variables included in the regression model was assessed using variance inflation factor (VIF) values. Localization status was defined as the dependent variable (0 = non-localized, 1 = localized). Model fit was assessed using the Hosmer–Lemeshow goodness-of-fit test, and model performance was evaluated using Nagelkerke *R*² value. Results were presented as odds ratio (OR) with 95% confidence interval.

The discriminative ability of iPTH levels to distinguish patients with successful localization was assessed using ROC (receiver operating characteristic) analysis, and the area under the curve (AUC) was calculated. The Youden Index criterion was used to determine the optimal cutoff value. The Youden Index was calculated by subtracting 1 from the sum of sensitivity and specificity (Youden Index = Sensitivity + Specificity − 1). The iPTH level yielding the highest Youden Index value was accepted as the optimal cutoff point.

Sample size calculation based on independent-­samples *t*-test with medium effect size (Cohen’s *d* = 0.5), alpha = 0.05, power = 0.80, and anticipated group ratio of 3 : 1 indicated a minimum requirement of 170 patients. All eligible patients (n = 174) meeting the inclusion criteria during the study period were included, resulting in 127 patients in the localized group and 47 patients in the non-localized group, ensuring adequate statistical power for the primary outcomes. Statistical analyses were performed using IBM SPSS Statistics for Windows, version 21.0 (IBM Corp.; Armonk, NY, USA). A *P*-value < .05 was considered statistically significant.

## Results

### Study Population and Descriptive Statistics

A total of 174 patients were included in the study. The descriptive statistics of patients’ demographic, biochemical, and clinical characteristics are shown in Table 1.

### Localization Success and Evaluation of Imaging Methods

Of the 174 patients included in the study, successful localization was achieved in 127 (73.0%) patients (localized group), while 47 (27.0%) patients could not be localized (non-localized group). The imaging results of both groups are presented in Table 2.

Parathyroid scintigraphy: Positive scintigraphy findings were observed in 120 of the 127 patients in the localized group (94.5%). All patients in the non-localized group had negative scintigraphy results. Positive scintigraphy findings were observed in the majority of patients in whom localization was achieved.

Neck USG: Positive ultrasonographic findings were present in 89 of the 127 patients (70.1%) in the localized group, whereas suspicious lesions were detected in 7 of the 47 patients (14.9%) in the non-localized group.

FNA-PTH washout: FNA-PTH washout was performed in selected patients with suspicious lesions detected on USG. In the localized group, washout evaluation was performed in 43 patients and yielded positive results in 37 (86.0%) cases. In 6 patients (14.0%), washout results were negative despite successful localization by scintigraphy.

In the non-localized group, PTH washout was performed in 7 patients with suspicious lesions detected on USG; all of these procedures yielded negative results.

Among patients in the localized group, scintigraphy was negative in 7 cases (5.5%), and localization in these patients was achieved through the combination of positive ultrasonography and positive PTH washout.

#### Parathyroid Adenoma Localization Distribution

When the parathyroid adenoma localizations of the 127 patients were evaluated, the most frequent localizations were the right lobe inferior pole (44.9%, n = 57) and the left lobe inferior pole (37.0%, n = 47). These two localizations constituted 81.9% of all cases. Other localizations included: right lobe superior pole 5.5% (n = 7), left lobe superior pole 5.5% (n = 7), mediastinal 2.4% (n = 3), intrathyroidal 3.1% (n = 4), ectopic 0.8% (n = 1), and bilateral 0.8% (n = 1).

#### Comparison of Localized and Non-localized Groups

The comparison of demographic and biochemical parameters between localized and non-localized groups is shown in Table 3. The significant differences between the groups were as follows: Serum calcium (*P *= .004) and iPTH levels (*P *= .0001) were higher in the localized group. Age was higher (*P *= .012), 25OHD levels were higher (*P *= .024), and the presence of thyroid nodules was more frequent (*P *= .016) in the non-localized group. No significant differences were observed between the groups in terms of other parameters.

**Figure 1:**
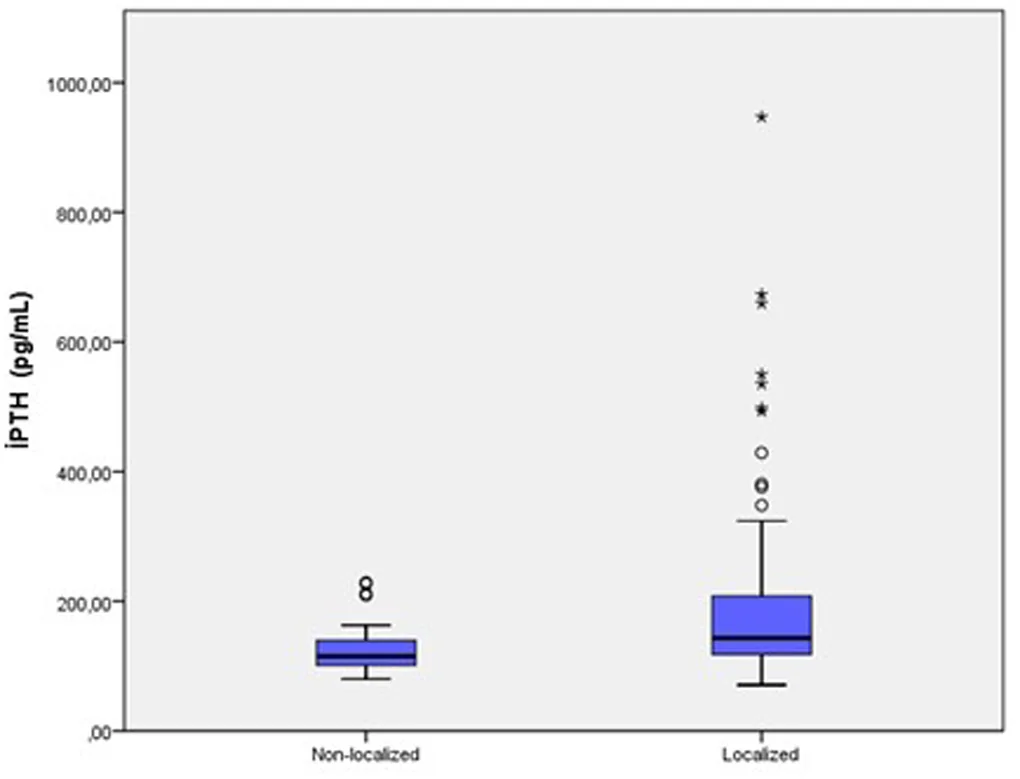
Boxplot showing serum intact parathyroid hormone (iPTH) levels in patients with localized and non-localized primary hyperparathyroidism based on conventional imaging.

A comparison of iPTH levels between localized and non-localized patient groups is presented in Figure 1. Patients in the localized group had significantly higher iPTH levels.

#### Clinical Management and Histopathological Findings

Of the 127 patients in the localized group, 106 (83.5%) underwent parathyroidectomy alone, while 21 (16.5%) underwent concomitant thyroid surgery. In the non-localized group, surgery was not performed because localization could not be achieved with conventional imaging, and therefore histopathological confirmation was unavailable.

Among patients who underwent concomitant thyroid surgery, benign thyroid pathology was observed in 16 cases, whereas thyroid ­malignancy was detected in 5 cases (3 papillary, 1 medullary, and 1 follicular thyroid carcinoma).

#### Factors Affecting Localization Success

Variables with *P *< .05 in univariate analyses (age, serum calcium, iPTH, 25OHD, and presence of thyroid nodules) were included in the logistic regression model. The multivariable logistic regression analysis is presented in Table 4. In multivariable logistic regression analysis, iPTH level (OR: 1.011; 95% CI: 1.002-1.020, *P *= .021) and presence of thyroid nodules (OR: 0.391; 95% CI: 0.174-0.879, *P *= .023) were identified as independently associated with localization success. The established model showed good statistical fit (Hosmer–Lemeshow test: *P *= .283). The Nagelkerke *R*² value of the established model was 0.260, indicating that the selected variables explained 26% of the variance in localization success. No evidence of significant multicollinearity was observed among the variables included in the model, with VIF values ranging from 1.052 to 1.334.

**Figure 2:**
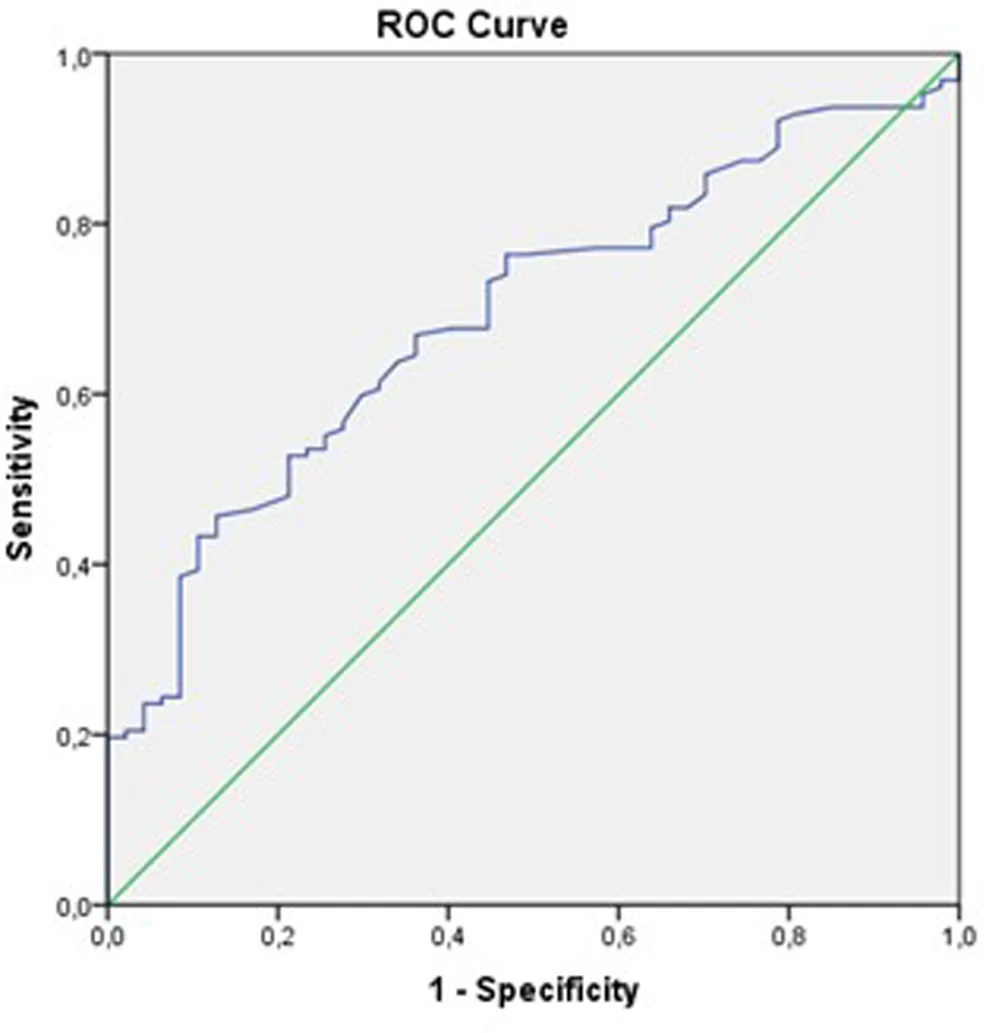
Receiver operating characteristic (ROC) curve demonstrating the performance of serum intact parathyroid hormone (iPTH) levels in predicting localization success with conventional imaging in primary hyperparathyroidism.

Receiver operating characteristic curve analysis was performed to evaluate the discriminative ability of iPTH levels for localization success (Figure 2). The analysis revealed an AUC of 0.693 for iPTH (95% CI: 0.611-0.774). This finding indicates that higher iPTH levels were associated with an increased likelihood of successful localization (*P *= .0001).

In ROC analysis, the optimal cut-off value for iPTH level was determined as 146.5 pg/mL according to the Youden Index criterion (Youden Index: 0.329, Sensitivity: 45.7%, Specificity: 87.2%). The ROC curve illustrating the association between iPTH levels and localization success is presented in Figure 2.

## Discussion

Accurate localization of hyperfunctioning parathyroid glands remains a key challenge in the management of PHPT. Despite advances in imaging modalities, localization remains a clinical problem in some patients. Neck USG and Tc99m-MIBI parathyroid scintigraphy are the primary imaging methods used for localization.^9^ These two methods have been frequently compared in the literature. In an observational cohort study, USG and Tc99m-MIBI showed similar performance.^10^ In the study by Kaur et al^11^, the sensitivity of USG (98%) was found to be higher than that of Tc99m-MIBI parathyroid scintigraphy (93%). Özkaya et al^12^ found that the sensitivity of Tc99m-MIBI parathyroid scintigraphy (85.3%) was higher than that of USG (72.5%). A meta-analysis showed similar localization performance between USG and Tc99m-MIBI SPECT in PHPT, with sensitivities of 76.1% vs 78.9% and positive predictive values of 93.2% vs 90.7%, respectively.^13^ Another method used for localization is ultrasound-guided FNA-PTH washout of the suspicious lesion. Its sensitivity for diagnosing PHPT has been found to be higher than that of Tc99-MIBI.^8^

In our study, 70.1% of patients in the localized group had positive USG findings, while 94.5% had positive scintigraphy results. Both imaging modalities contributed to parathyroid localization; however, positive scintigraphy findings were more commonly observed in patients with successful localization compared with ultrasonography. This difference may be attributed to the combination of Tc99m-MIBI parathyroid scintigraphy with SPECT/CT in our patients under study, the inability of USG to detect mediastinal (2.4%) and ectopic (0.8%) lesions, and the potential misinterpretation of intrathyroidal parathyroid lesions (3.1%) as thyroid nodules. The higher rate of positive scintigraphy findings observed in our cohort may be related to the functional characteristics of Tc99m-MIBI imaging and the limitations of ultrasonography in detecting mediastinal or ectopic lesions.

Negative PTH washout results were observed in 6 patients in the localized group. This may be attributable to operator-dependent factors such as technical variability or level of experience. In 5.5% (7/127) of the localized group, scintigraphy was negative, and localization was achieved only through positive USG and PTH washout. In 30 patients within the localized group, PTH washout was performed despite positive scintigraphy findings. This approach was adopted to confirm localization, particularly in the presence of concomitant thyroid nodules, due to the potential for false-positive Tc99m-MIBI results.^14^

Among the 127 patients who could be localized, the most frequent localizations were the right lower lobe (44.9%) and left lower lobe (37.0%), with 81.9% of cases located in the lower poles. The more frequent occurrence in the lower poles was consistent with the literature.^15^^,^^16^ While the superior parathyroid glands originate from the fourth pharyngeal pouch, the inferior parathyroid glands originate from the third pharyngeal pouch together with the thymus gland.^17^ Adenoma development may be more frequent in these glands due to the relatively longer and more complex embryological migration pathways of the inferior parathyroid glands, their greater anatomical variations, and their relationship with the thymus.

This study aimed to identify factors affecting the success of imaging-based localization, univariate analysis revealed that among laboratory parameters, elevated calcium and PTH levels and low vitamin D levels were associated with localization. Advanced age was statistically significant in the non-localized group. In multivariable logistic regression analysis, iPTH level and the presence of thyroid nodules were independently associated with localization success. Our findings suggest that localization success in PHPT may be influenced not only by biochemical disease severity but also by concomitant thyroid pathology. In particular, patients with relatively lower PTH levels and coexisting thyroid nodules may represent a distinct subgroup with a higher risk of negative localization using conventional imaging modalities.

The concomitant thyroid nodule rate in PHPT varies between 15% and 75% according to the literature, while the incidence of thyroid malignancy ranges from 2% to 29.8%.^18^^-^^20^ The nodule occurrence rate in PHPT is currently similar to the rate of up to 70% observed in the normal population with the widespread use of neck USG, but the malignancy rate is higher than that in the normal population.^21^ In our study, 59.8% of patients had thyroid nodules. Thyroid malignancy was detected in 5 (23.8%) of the 21 patients who underwent total thyroidectomy and lobectomy.

Various studies in the literature demonstrate discordance in localization between Tc99m-MIBI parathyroid scintigraphy and neck USG in cases of PHPT with concomitant thyroid disease.^22^^,^^23^ Medas et al^24^ also demonstrated that the sensitivity of USG and Tc99m-MIBI scintigraphy decreases in the presence of concomitant thyroid pathologies. In a prospective study conducted by Berber et al^25^, the presence of concomitant thyroid disease was found to adversely affect localization by both USG and Tc99m-MIBI (*P* = .0013, *P* = .0159, respectively). Contrary to these studies, there are also data showing that the presence of thyroid nodules does not reduce the sensitivity of imaging methods.^26^ In our study, it was found that the localization success rate decreased by 60.9% in patients with concomitant thyroid nodules compared to those without (OR: 0.391, 95% CI: 0.174-0.879, *P* = .023). This may be due to posterior displacement of the adenoma by the enlarged thyroid on USG, poor sonographic penetration through thyroid tissues, or difficulty in distinguishing from adjacent nodular thyroid contour.^27^ These findings suggest that localization success in PHPT may depend not only on biochemical disease severity but also on anatomical factors such as concomitant thyroid pathology. However, information regarding thyroid volume, multinodular nodule burden, and detailed sonographic characteristics was not systematically available in our dataset; therefore, residual confounding cannot be excluded.

Primary hyperparathyroidism most commonly occurs in the 50-60 age range and affects 2% of the population aged 55 and older.^14^ In our , the mean age of patients was 57.45 ± 12.50, which was consistent with the literature. In several studies examining the effect of age factor on localization, no significant difference was found.^24^^,^^25^ In a retrospective study, patients who could not be localized by imaging methods were found to be older, but no statistically significant difference was detected.^28^ Mathew et al^23^ reported discordance in localization methods in elderly patients. The loss of significance of the age factor in multivariable analysis in our study suggests that the effect of age on localization difficulty may be mediated through concomitant thyroid pathology or other factors.

Various studies in the literature have examined biochemical parameters associated with localization detected by imaging methods. Zhu et al^28^ demonstrated that the sensitivity of SPECT/CT is related to serum PTH concentration. While the sensitivity was 84.72% in patients with PTH levels ≤252 pg/mL, it was found to be 95.00% in the high PTH group (*P* = .022). No significant difference was observed between calcium, ALP, creatinine, 25OHD levels, and localization.^28^ In a study by Cordes et al^2^^9^, PTH level was found to be associated with Tc99m-MIBI localization (*P* = .049), and iPTH values of Tc99m-MIBI-negative adenomas were found to be below 150 pg/mL. They reported that calcium and 25-OH vitamin D levels were not significantly associated with Tc99m-MIBI localization. In another study, PTH values below 160 pg/mL and serum calcium values below 11.3 mg/dL were shown to reduce the chance of localization with Tc99m-MIBI.^30^ Berber et al^25^ also found that increased serum PTH and calcium levels were associated with localization by USG and Tc99m-MIBI. In another retrospective study, a significant relationship was found between ionized calcium, phosphate, PTH levels, and the positivity of Tc99m-MIBI SPECT/CT scanning.^31^

In our study, higher iPTH levels were associated with successful localization, which is generally consistent with previous studies evaluating biochemical factors related to imaging outcomes in PHPT. ROC analysis demonstrated moderate discriminative ability for iPTH (AUC = 0.693), with high specificity (87.2%) but relatively low sensitivity (45.7%) at the cut-off value of 146.5 pg/mL. Therefore, although iPTH may contribute to the interpretation of localization outcomes, its value as a standalone clinical marker appears limited.

Based on our findings, patients with lower iPTH levels, particularly in the presence of concomitant thyroid nodules, may require more cautious interpretation of conventional imaging findings. In selected cases, additional imaging modalities such as 4-dimensional computed tomography (4D-CT) or fluorocholine positron emission tomography with computed tomography (PET/CT) may be considered as part of individualized clinical evaluation. While several previous studies have evaluated the relationship between biochemical parameters and localization success, our study additionally highlights the potential interaction between biochemical severity and concomitant thyroid pathology. This combined effect may help explain negative localization results observed in a subset of PHPT patients despite the use of standard imaging modalities.

As mentioned previously, there are publications in the literature that found no significant relationship between 25OHD levels and localization.^28^^29^^,^^32^^,^^33^ In one of these studies, it was found that combining MIBI scan results with high PTH levels (twice the mean value) in patients with low 25OHD levels (<50 nmol/L) increased scanning specificity from 80% to 93%. Therefore, Tc99m-MIBI specificity increases in cases of concomitant vitamin D deficiency in PHPT.^14^^,^^33^ In our study, vitamin D and calcium levels lost statistical significance in multivariable analysis despite being significant in univariate analysis. This finding may reflect interactions between vitamin D status, PTH secretion dynamics, and disease severity rather than a direct independent effect of vitamin D on localization outcomes. The underlying mechanism remains unclear and should be interpreted cautiously given the loss of significance in multivariable analysis.

There are studies examining the relationship of localization in PHPT with many factors such as lesion size, weight, and oxyphil cell content. Different parathyroid cell types, which are thought to represent different phases of the cell cycle, affect the accuracy of MIBI scanning. The MIBI uptake ability of each cell type has been associated with mitochondrial content. Chief and oxyphil cells are rich in mitochondria and show greater MIBI uptake, while clear and fat cells have low mitochondrial content and lower MIBI uptake.^14^^,^^24^

Based on these findings, patients with relatively low PTH levels and concomitant thyroid nodules may represent a subgroup at higher risk for unsuccessful localization using conventional imaging modalities.

The limitations of the study include the inability to establish a clear cause-and-effect relationship due to its retrospective and single-center nature, limited generalizability of results, and inability to reflect practices at different centers. Because surgery was not performed in the non-localized group, histopathological confirmation of the underlying parathyroid pathology could not be obtained. Although the diagnosis of PHPT in the non-localized group was established biochemically according to international guidelines, the lack of surgical confirmation remains an important limitation. As a result, it was not possible to distinguish true localization failure from unsuccessful localization using conventional imaging with certainty, and the exact pathological etiology (such as adenoma or multiglandular disease) could not be determined in these patients, which may have introduced potential misclassification and verification bias. Because histopathological confirmation could not be performed in the non-localized group, the true sensitivity and specificity values of imaging methods could not be calculated. Follow-up imaging and long-term clinical outcome data were not consistently available for the non-localized group, which limited further assessment of their subsequent clinical course and diagnostic confirmation. In addition, FNA-PTH washout was performed selectively rather than systematically in all patients, which may have introduced heterogeneity into the localization status.

Due to incomplete histopathology data, size- and weight-related analyses could not be performed. Because the study was retrospective, standardization of imaging evaluations could not be ensured. Furthermore, ultrasonography is an operator-dependent imaging modality, and subjective differences between operators may have influenced localization outcomes. In addition, bone mineral density was evaluated using lumbar spine and femoral neck measurements, whereas forearm (distal radius) measurement, which is recommended in patients with PHPT due to predominant cortical bone involvement, was not routinely available in our dataset. Because the study was cross-sectional and retrospective, long-term surgical success and recurrence rates cannot be evaluated. Comparison with new imaging techniques currently used for localization (4D-CT, PET/CT) could not be performed. Furthermore, advanced imaging modalities such as 4D-CT or PET/CT were not systematically performed in the non-localized group; therefore, it remains uncertain whether localization can be achieved using more sensitive imaging techniques in these patients.

Accurate localization plays a key role in the management of PHPT. This study evaluated the factors affecting localization success in patients with PHPT and identified clinical characteristics associated with unsuccessful localization. In the evaluated patient population, concomitant thyroid nodules and PTH levels were the factors most strongly associated with localization outcomes. While higher iPTH levels were associated with localization success, the presence of concomitant thyroid nodules was associated with a lower likelihood of localization. These findings may help clinicians better understand factors associated with unsuccessful localization using conventional imaging in PHPT.

## Figures and Tables

**Table 1.** Descriptive Statistics of Patients Demographic, Biochemical, and Clinical Characteristics

Variable	Patients (N = 174)
Demographic characteristics	
Age (years), mean ± SD	57.45 ± 12.50
Gender, n (%)	
Female	151 (86.8)
Male	23 (13.2)
Biochemical parameters	
Serum calcium (mg/dL), median (min-max)	11.45 (10.7-14.5)
Serum phosphate (mg/dL), mean ± SD	2.64 ± 0.54
25 hydroxyvitamin D (ng/mL), median (min-max)	18.55 (4.0-90.0)
iPTH (pg/mL), median (min-max)	136.00 (71.0-947.0)
24-hour urine calcium (mg/day), median (min-max)	300.50 (92.0-790.0)
eGFR (mL/min/1.73m²), mean ± SD	82.09 ± 18.48
Alkaline phosphatase (U/L), median (min-max)	90.00 (32.0-304.0)
Clinical characteristics, n (%)	
DEXA results	
Normal	43 (24.7)
Low bone mass	60 (34.5)
Osteoporosis	71 (40.8)
Urinary stones	41 (23.6)
Thyroid nodules	104 (59.8)
Hypothyroidism	4 (2.3)
Hyperthyroidism	5 (2.9)
Positive imaging findings, n (%)	
Neck ultrasonography	96 (55.2)
Parathyroid scintigraphy	120 (69.0)

DEXA, dual-energy X-ray absorptiometry; eGFR, estimate glomerular filtration rate; iPTH, parathyroid hormone intact.

**Table 2.** Distribution of Imaging Findings in Patients with Localized and Non-Localized Disease

Localization Method	Localized Group (n = 127), n (%)	Non-localized Group (n = 47), n (%)	Total (N = 174), n (%)
Neck ultrasonography			
Negative	38 (29.9)	40 (85.1)	78 (44.8)
Positive	89 (70.1)	7 (14.9)	96 (55.2)
Parathyroid scintigraphy			
Negative	7 (5.5)	47 (100.0)	54 (31.0)
Positive	120 (94.5)	0 (0.0)	120 (69.0)
FNA-PTH washout (n = 50)	(n = 43)	(n = 7)	(n = 50)
Negative	6 (14.0)	7 (100.0)	13 (26.0)
Positive	37 (86.0)	0 (0.0)	37 (74.0)

FNA, fine-needle aspiration; PTH, parathyroid hormone.

**Table 3.** Comparison of Demographic and Biochemical Parameters Between Localized and Non-localized Groups

Variable	Localized Group (n = 127)	Non-localized Group (n = 47)	*P*
Demographic characteristics			
Age (years), mean ± SD	56.00 ± 12.27	61.36 ± 12.41	.012*
Gender (Female), n (%)	108 (85.0)	43 (91.5)	.265
Biochemical parameters			
Serum calcium (mg/dL), median (min-max)	11.6 (10.7-14.5)	11.2 (10.7-13.5)	.004*
Serum phosphate (mg/dL), mean ± SD	2.61 ± 0.54	2.72 ± 0.53	.211
25- hydroxyvitamin D (ng/mL), median (min-max)	17.0 (4.0-56.0)	21.8 (4.8-90.0)	.024*
iPTH (pg/mL), median (min-max)	143.0 (71.0-947.0)	115.0 (80.0-229.0)	<.001*
24-hour urine calcium (mg/day), median (min-max)	307.0 (92.0-790.0)	279.0 (115.0-610.0)	.340
eGFR (mL/min/1.73m²), mean ± SD	83.23 ± 18.73	79.00 ± 17.60	.181
Alkaline phosphatase (U/L), median (min-max)	90.0 (32.0-304.0)	90.0 (42.0-204.0)	.337
Clinical characteristics, n (%)			
DEXA results			
Normal	33 (26.0)	10 (21.3)	.587
Low bone mass	41 (32.3)	19 (40.4)	
Osteoporosis	53 (41.7)	18 (38.3)	
Urinary stones, n (%)			
Negative	95 (74.8)	38 (80.9)	.404
Positive	32 (25.2)	9 (19.1)	
Thyroid nodules, n (%)			
Negative	58 (45.7)	12 (25.5)	.016*
Positive	69 (54.3)	35 (74.5)	
Hypothyroidism, n (%)			
Negative	124 (97.6)	46 (97.9)	.927
Positive	3 (2.4)	1 (2.1)	
Hyperthyroidism, n (%)			
Negative	125 (98.4)	44 (93.6)	.092
Positive	2 (1.6)	3 (6.4)	

* P < .05 iPTH: parathyroid hormone intact, eGFR: estimate glomerular filtration rate, DEXA: dual-energy X-ray absorptiometry.

**Table 4.** Multivariable Logistic Regression Analysis of Factors Affecting Localization Success in Primary Hyperparathyroidism

Variable	B	S.E.	Wald	*P*	OR	95% CI
Age (years)	−0.029	0.018	2.770	.096	0.971	0.938-1.005
25-OH Vitamin D (ng/mL)	−0.019	0.017	1.326	.249	0.981	0.950-1.014
Serum calcium (mg/dL)	0.653	0.349	3.497	.061	1.921	0.969-3.809
iPTH (pg/mL)	0.011	0.005	5.336	.021*	1.011	1.002-1.020
Thyroid nodules	−0.938	0.413	5.170	.023*	0.391	0.174-0.879
Constant	−5.335	3.861	1.909	.167	0.005	

CI, confidence interval; iPTH, intact parathyroid hormone; OR, odds ratio; S.E., standard error.

*P < .05.

## Data Availability

The data that support the findings of this study are available on request from the corresponding author.
